# Soybean TIP Gene Family Analysis and Characterization of GmTIP1;5 and GmTIP2;5 Water Transport Activity

**DOI:** 10.3389/fpls.2016.01564

**Published:** 2016-10-21

**Authors:** Li Song, Na Nguyen, Rupesh K. Deshmukh, Gunvant B. Patil, Silvas J. Prince, Babu Valliyodan, Raymond Mutava, Sharon M. Pike, Walter Gassmann, Henry T. Nguyen

**Affiliations:** ^1^Division of Plant Science, National Center for Soybean Biotechnology, University of MissouriColumbia, MO, USA; ^2^Departement de Phytologie, Laval UniversityQuebec, QC, Canada; ^3^Division of Plant Sciences and Interdisciplinary Plant Group, Christopher S. Bond Life Sciences Center, University of MissouriColumbia, MO, USA

**Keywords:** aquaporin, tonoplast intrinsic proteins (TIPs), water transporter, soybean, abiotic stress, expression, SNP

## Abstract

Soybean, one of the most important crops worldwide, is severely affected by abiotic stress. Drought and flooding are the major abiotic stresses impacting soybean yield. In this regard, understanding water uptake by plants, its utilization and transport has great importance. In plants, water transport is mainly governed by channel forming aquaporin proteins (AQPs). Tonoplast intrinsic proteins (TIPs) belong to the plant-specific AQP subfamily and are known to have a role in abiotic stress tolerance. In this study, 23 soybean TIP genes were identified based on the latest soybean genome annotation. TIPs were characterized based on conserved structural features and phylogenetic distribution. Expression analysis of soybean TIP genes in various tissues and under abiotic stress conditions demonstrated tissue/stress-response specific differential expression. The natural variations for TIP genes were analyzed using whole genome re-sequencing data available for a set of 106 diverse soybean genotypes including wild types, landraces and elite lines. Results revealed 81 single-nucleotide polymorphisms (SNPs) and several large insertions/deletions in the coding region of TIPs. Among these, non-synonymous SNPs are most likely to have a greater impact on protein function and are candidates for molecular studies as well as for the development of functional markers to assist breeding. The solute transport function of two TIPs was further validated by expression in *Xenopus laevis* oocytes. GmTIP1;5 was shown to facilitate the rapid movement of water across the oocyte membrane, while GmTIP2;5 facilitated the movement of water and boric acid. The present study provides an initial insight into the possible roles of soybean TIP genes under abiotic stress conditions. Our results will facilitate elucidation of their precise functions during abiotic stress responses and plant development, and will provide potential breeding targets for modifying water movement in soybean.

## Introduction

The need for sustainable production of food is a critical issue for human and environmental health due to the continuously growing global population. Soybean, being a source of edible oil and protein rich meal, is considered a promising crop to fulfill the increasing food demand (Krishnamurthy and Shivashankar, 1975). Soybean seed contains over 40% protein and 20% oil. However, stresses imposed by environmental factors greatly affect soybean yield and quality. Water is one of the important factors causing severe yield losses either with excess availability from flooding or with limitation resulting from drought. Plants combat such stresses by regulating water distribution at different levels, such as the vascular system and the permeability of plasma membranes. Aquaporins (AQPs), a class of channel forming proteins, facilitate transport of water and many other solutes across cellular membranes (Javot and Maurel, 2002; Maurel et al., 2002; Tyerman et al., 2002). AQPs are integral membrane proteins that belong to the major intrinsic protein (MIP) family. In higher plants, MIPs are classified into five subfamilies, including plasma membrane intrinsic proteins (PIPs), tonoplast intrinsic proteins (TIPs), NOD 26-like intrinsic proteins (NIPs), small basic intrinsic proteins (SIPs), and uncategorized intrinsic proteins (XIPs) (Chaumont et al., 2001; Kaldenhoff and Fischer, 2006; Danielson and Johanson, 2008).

It has been shown that the expression of TIP genes varies in different tissue, hormone and abiotic treatments. *ZmTIP2-3* transcripts was detected only in maize root tissues and was induced by salt and water stresses (Lopez et al., 2004). Cotton *GhTIP1:1* transcripts mainly accumulated in roots and hypocotyls under normal conditions, but were dramatically up-regulated in cotyledons and down-regulated in roots within a few hours after cotton seedlings were cold-treated (Li et al., 2009). Several root-specific *RB7-type TIP* genes have been identified from *Arabidopsis thaliana* (Yamamoto et al., 1990), *Solanum tuberosum* (Heinrich et al., 1996), *Petroselinum crispum* (Roussel et al., 1997), *Helianthus annuus* (Sarda et al., 1999), *Mesembryanthemum crystallinum* (Kirch et al., 2000). Recently, one strawberry *RB7-type TIP* gene, *FaRB7* also exhibited a root-specific expression pattern (Vaughan et al., 2006).

Tonoplast intrinsic proteins genes also have been reported to be involved in the elevation of abiotic stress tolerance in several plant species. Notably, the TIP gene *TsTIP1;2* from *Thellungiella salsuginea* provided increased tolerance against drought, salt and oxidative stresses when ectopically expressed in *Arabidopsis* (Wang et al., 2014). Similarly, increased salinity tolerance was achieved with the heterologous expression of tomato *SITIP2;2* in *Arabidopsis* (Xin et al., 2014). In soybean, Zhang et al. (2016) observed that the expression pattern of *GmTIP2;3* was affected by PEG and ABA, and overexpressing *GmTIP2;3* in yeast cells improved osmotic stress tolerance. However, another TIP2 gene, *GsTIP2;1* cloned from *Glycine soja*, resulted in reduced tolerance to salt and dehydration stress when overexpressed in *Arabidopsis* (Wang et al., 2011). Such contrasting results indicate diverse regulation of TIPs within the subfamily that may be due to tissue-specific expression patterns, since *GmTIP2;3* was found to be highly expressed in roots whereas *GsTIP2;1* showed comparatively higher expression in leaves. Higher water movement by *GmTIP2;3* in roots can be correlated with efficient water uptake, leading to enhanced osmotic stress tolerance. In contrast, water movement regulated by *GsTIP2;1* seems to increase water loss through transpiration. Similar observations have been reported in rice, where unbalanced expression of most aquaporins in leaves compared to root is thought to result in rapid depletion of leaf water and subsequent inhibition of photosynthesis (Nada and Abogadallah, 2014).

Slow wilting is an important physiological parameter to study water stress tolerance in soybean. Genetic variation observed for the slow wilting trait has proven very useful for improving yield under drought conditions (Sloane et al., 1990). Several genotypes display heritable variation for the slow wilting phenotype. For instance, Sloane et al. (1990) observed delayed wilting in PI 416937 and PI 471938 soybean lines under drought conditions in the field. Recently, two new genotypes, PI 567731 and PI 567690, have been identified for the slow wilting trait under field conditions (Pathan et al., 2014). Interestingly, a study conducted with slow wilting soybean line PI416937 has revealed association between AQPs and hydraulic conductance (Sadok and Sinclair, 2010a,b, 2012). Devi et al. (2015) reported down-regulation of AQPs under high vapor pressure deficit (VPD) conditions in PI 416937, which may be due to the reduced uptake of water, resulting in conservation for later availability. Recently, our study has identified several differentially expressed AQP genes among the slow wilting and fast wilting soybean lines (Prince et al., 2015). These results prompted us to undertake detailed studies of candidate AQPs thought to be involved in soybean abiotic stress tolerance. (Verkman et al., 2014; Madeira et al., 2016).

In the present study, a comprehensive analysis of the soybean TIP gene family was carried out including phylogenetic relationships, chromosomal location, gene duplication status, gene structure, conserved motif, expression profiling under abiotic stress, and natural variation in soybean wild types, landraces and elite lines. The water transport function of two GmTIP proteins was validated through oocyte experiments. *Xenopus laevis* oocytes have very low background activity that helps to achieve a high signal-to-noise ratio as required to study transporters. Therefore, *Xenopus* oocytes have been routinely used for the evaluation of solute permeability by several different transporters (Osawa et al., 2006; Verkman et al., 2014). More particularly, AQPs are frequently analyzed using *X. laevis* oocytes (Maurel et al., 1993; Deshmukh et al., 2015). These data will contribute to future studies to functionally characterize TIP proteins in soybean.

## Materials and Methods

### Identification and Structural Organization of Tonoplast Intrinsic Protein (TIP) Genes

The *Arabidopsis* TIP2;1 amino acid sequence was used as query to perform a database search using BLASTP against predicted proteins in the *G. max Wm82.a2.v1* genome derived from Phytozome databases. Sequence with at least 50% identity with the query sequence was classified as candidate GmTIPs. BLAST hits with less than a 100 bitscore were removed. Manual curation was then performed to match TIPs identified in the present study with those reported earlier by Deshmukh et al. (2013). The genomic sequences, CDS, and protein sequences for all GmTIPs were retrieved from Phytozome (V11^1^). Novel TIP genes identified with the recent version of the soybean genome annotation were characterized for the aromatic/arginine (Ar/R) selectivity filters (SFs), Froger’s residues, Asn-Pro-Ala (NPA) motifs and the spacing between NPAs. The exon/intron organizations of GmTIPs was visualized with the Gene Structure Display Server program (Hu et al., 2015; GSDS^2^).

### Identification of Conserved Protein Motif and Channel Structure Prediction

The protein sequences were analyzed to identify conserved protein motifs (Motif scan) using the ‘Multiple EM for Motif Elicitation’ (MEME) program (Bailey et al., 2006). Transmembrane domains in newly identified TIPs were predicted using TOPCONS software tools^3^. Protein structures were modeled based on the structure of *Arabidopsis* TIP2;1^4^. The Pore Walker tool was used to predict the pore feature^5^ (Pellegrini-Calace et al., 2009).

### Phylogenetic Tree Analysis

Multiple sequence alignments were conducted with the amino acid sequence of GmTIPs. Subsequently, a phylogenetic tree was constructed using the Maximum-Likelihood method provided in the MEGA 6.0 software tool (Tamura et al., 2013). The reliability of an inferred tree was confirmed with bootstrap analysis performed with 1,000 replications.

### Chromosomal Distribution and Gene Duplications in GmTIPs

The chromosomal location of all GmTIP genes was obtained through BLASTN searches against the *G. Max Wm82.a2.v1* genome database in Phytozome. GmTIPs were located on soybean chromosomes based on physical positions. To further analyze gene duplication events, synonymous substitution (Ks) and non-synonymous substitution (Ka) rates were downloaded from the Plant Genome Duplication Database (PGDD) database^6^. The date of duplication events was subsequently estimated according to the equation *T* = Ks/2λ, in which the mean synonymous substitution rate (λ) for soybean is 6.1 × 10^-9^ (Lynch and Conery, 2000).

### Expression Profiling Using RNA-seq Datasets

The RNA-seq data generated by Libault et al. (2010) for nine different tissues including flower, leaves, nodules, pod, root, root hair, seed, shoot apical meristem, and stem were used to analyze expression patterns of GmTIPs. Expression profiling of GmTIPs in leaf tissues of PI 567690 (drought tolerant) and Pana (drought susceptible) grown under drought conditions, and PI 408105A (flooding tolerant) and S99-2281 (PI 654356, flooding susceptible) grown under flooding conditions, was extracted from earlier reported data (Mutava et al., 2015; Prince et al., 2015; Syed et al., 2015). Briefly, at the V5 stage (five unfolded trifoliate leaves), drought stress was imposed by withdrawing water for 21 days and flooding stress was imposed by overwatering for 15 days. Similarly, RNA-seq data for Williams 82 plants subjected to very mild stress (VMS), mild stress (MS), and severe stress (SS) conditions, as well as recovery from severe stress after re-watering (SR), was used to study expression of GmTIPs (Song et al., 2016). Hierarchical clustering of expression data was performed using dCHIP software (Li and Wong, 2001).

### Analysis of Synonymous and Non-synonymous SNP Variants in 106 Soybean Lines

All single-nucleotide polymorphism (SNP) datasets located in exonic regions were extracted from whole genome re-sequencing (sequencing depth approximately 15X) data (Valliyodan et al., 2016) as described by Patil et al. (2016). Annotation and effect prediction for the SNPs and other variants were performed using SnpEff^7^ SNPs were further classified into synonymous and non-synonymous categories.

### *Xenopus laevis* Oocyte Assay

Full length cDNAs of soybean aquaporins were cloned into the *Xenopus* expression vector pOO2, which contains 5′ and 3′ UTR regions of the *X. laevis* β-globin gene, including an extended polyA tract for improved mRNA stability and expression (Ludewig et al., 2002). cRNA was synthesized using the SP6 Ambion mMessage mMachine kit with linearized pOO2 constructs as templates. Oocytes were isolated and maintained as described (Pike et al., 2009) and injected with 46 ng of cRNA per oocyte. Water transport was evaluated by perfusing oocytes with hypoosmotic ND96 solution and monitoring swelling over time (Maurel et al., 1993; Durbak et al., 2014). In addition, boron transport was evaluated by replacing the NaCl-containing ND96 with an isoosmotic boron-ND96 solution and monitoring swelling over time (Durbak et al., 2014).

## Results

### Genome-Wide Identification of Tonoplast Intrinsic Proteins in Soybean

A total of twenty-three TIP genes was identified based on a comprehensive phylogenetic tree analysis within the GmMIP gene family (Deshmukh et al., 2013; Zhang et al., 2013). Here TIP genes were reanalyzed based on a recently released version of the soybean genome annotation (Wm82.a2.v1). The number of TIPs identified in soybean is in good agreement with earlier studies performed with a previous genome annotation (Deshmukh et al., 2013; Zhang et al., 2013). However, the transcript of *GmTIP4;2* (*Glyma04G08830*) reported in earlier studies seems to have been based on a mispredicted gene model and was therefore removed from the Wm82.a2.v1 annotation. Another TIP gene, *Glyma12g01490*, earlier reported as a pseudo-gene because of transcript truncation, was determined to generate a full-length transcript (new ID *Glyma.12G012300*) in the new version. Two other TIP pseudogenes reported in earlier studies were not present in the new genome version. Therefore, one gene was removed and one added, resulting in a corrected set of 23 TIPs. The gene names, gene IDs and locations in the genome are listed in Supplementary Table S1. The newly identified TIP gene (*Glyma12G01490*) was named *GmTIP5;2* according to the clustering in the phylogenetic tree.

### Phylogenetic Relationship and Gene Structure

**FIGURE 1:**
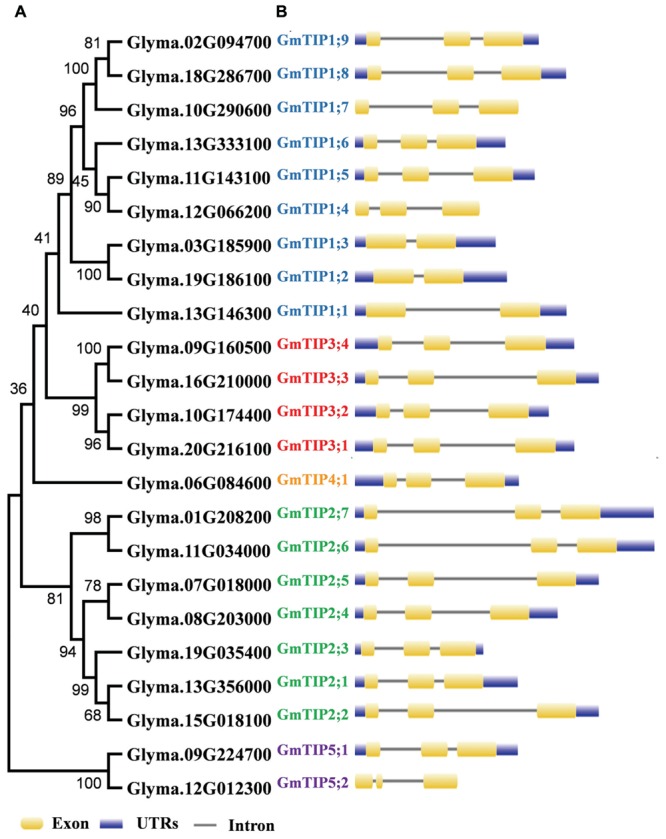
**Phylogenetic relationship and exon-intron structure of soybean Tonoplast intrinsic proteins (TIPs). (A)** The unrooted tree was constructed via alignment of full-length amino acid sequences from soybean using MEGA6 software by the neighbor-joining method. **(B)** Lengths of the exons and introns of each TIP gene are displayed proportionally. Exons and introns are indicated by yellow rectangles and thin lines, respectively. The untranslated regions (UTRs) are indicated by blue rectangles.

To get a better understanding of the evolutionary history, an unrooted phylogenetic tree was constructed using the Maximum-Likelihood (ML) method on the basis of multiple sequence alignment of the 23 soybean TIP proteins (**Figure 1A**). According to the ML phylogenetic tree, the TIP family is divided into five subgroups designated as Group 1–Group 5. Group 1, the largest clade, contains nine members, representing 39.1% of the total TIP genes. Group 4 constitutes the smallest clade with only one member. To gain further insights into the evolutionary relationships among GmTIP genes, the exon/intron structures of individual GmTIP genes were predicted based on the alignment of CDS sequences with corresponding genomic DNA sequences. As illustrated in **Figure 1B**, 20 out of 23 GmTIP genes have three exons, while the remaining three only possess two exons. Genes within the same clade demonstrated similar exon/intron distribution patterns in terms of exon/intron length, with the exception of GmTIP5;2 containing a short second exon.

### Chromosomal Location and Duplication of Soybean TIP Genes

**FIGURE 2:**
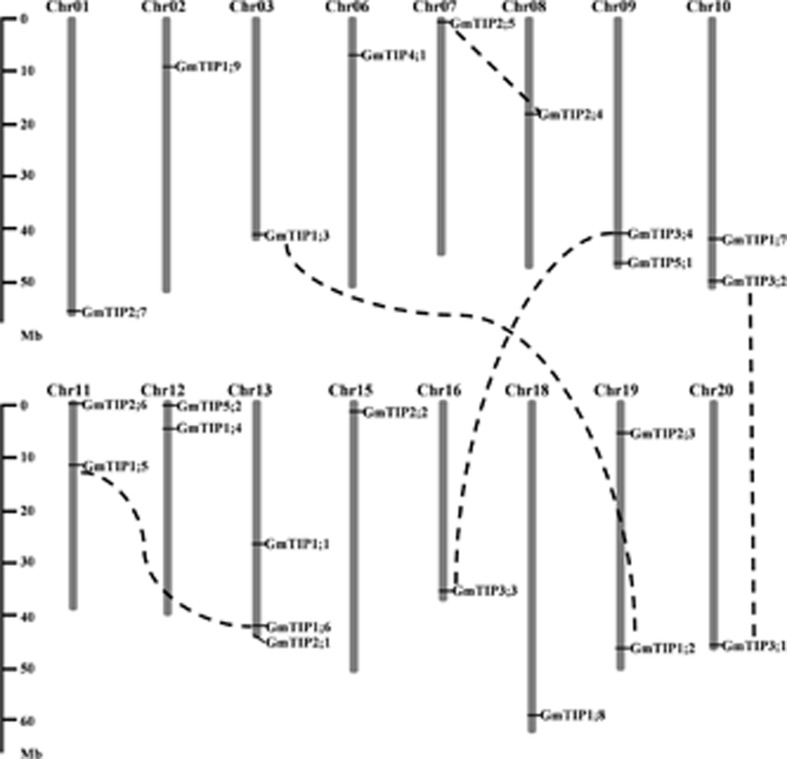
**Chromosomal locations and gene duplication events of soybean TIP gene family members.** The segmentally duplicated gene pairs are linked by black dotted lines. The left scale represents physical distance along the chromosomes in megabases (Mb). Chromosome numbers are shown at the top of each vertical gray bar.

The 23 TIP genes were unevenly distributed on 16 of the 20 soybean chromosomes (**Figure 2**), with three GmTIPs on chromosome 13, and two GmTIPs each on chromosomes 9, 10, 11, 12, and 19. The remaining chromosomes only contained one TIP each.

During evolution, the soybean genome has undergone two rounds of whole genome duplication (Schmutz et al., 2010). In order to examine the duplication patterns of soybean TIP genes, the PGDD was searched to identify segmentally duplicated pairs, and tandem duplication was identified based on the gene loci. No tandem duplication was found in this gene subfamily. To identify duplicated pairs, synonymous (Ks) and non-synonymous substitution (Ka) distance values were calculated and the Ka/Ks ratios were used to evaluate the duplication time. The Ka/Ks ratio for each segmentally duplicated gene pair varied from 0.06 to 0.28 (Supplementary Table S2). This analysis suggests that all mutations in paralogous GmTIP genes are neutral or disadvantageous, as their Ka/Ks ratios were less than 1. We found that the five closest branches of soybean TIP genes experienced duplication during the soybean whole genome duplication period, while the others were duplicated 67 Mya or earlier. The duplicated GmTIPs exist in the form of sister pairs in the phylogenetic tree (**Figure 1**) and are shown linked by dotted lines in **Figure 2**.

### Sequence and Conserved Domain Analysis in GmTIP Gene Family

The protein size of TIP members varied from 238 to 256 amino acids with 78–99% identity in each subgroup. All soybean GmTIP proteins contain two NPA motifs, and the amino acids representing the Ar/R selectivity filter (SF) and Froger’s residues are highly conserved. The Ar/R SF in the newly identified GmTIP5;2 consists of the amino acid sequence S-V-G-C, and the spacing between the NPA domains is 110 amino acids, which is consistent with other members of the GmTIP5 subfamily (Supplementary Table S3). Similarly, the sequence A-A-Y-W is a common feature for both GmTIP5;1 and GmTIP5;2.

**FIGURE 3:**
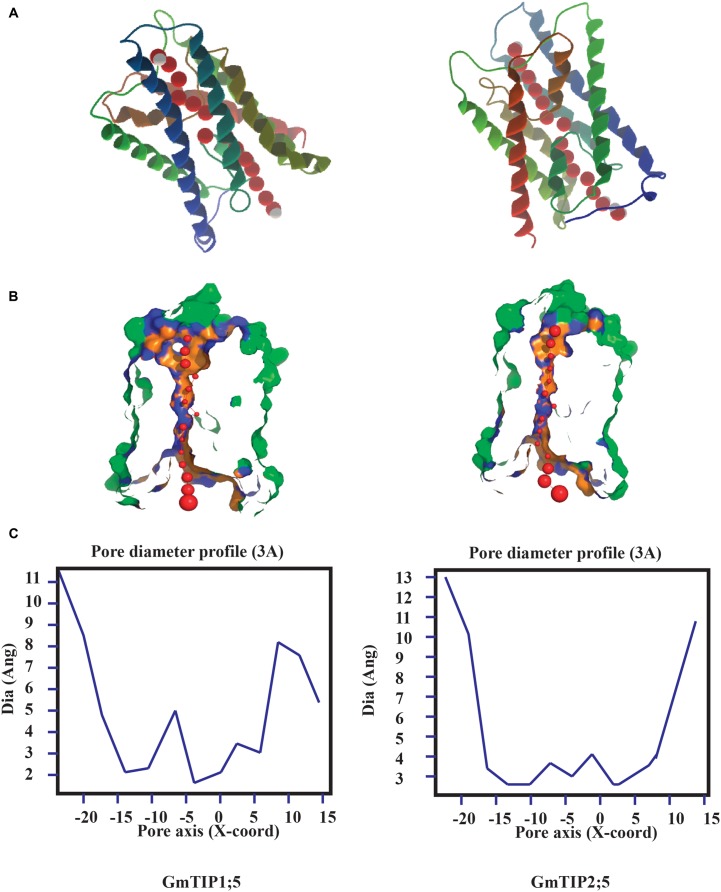
**Protein tertiary structure showing pore morphology of GmTIP1;5 and GmTIP2;5. (A)** Tertiary structures comprised of six transmembrane domains and water molecules (red) passing through the pores of GmTIP1;5 (left) and GmTIP2;5 (right) visualized with CLC genomic workbench. **(B)** Cross sections of the proteins showing pore. **(C)** Pore diameter profile of GmTIP1;5 (left) and GmTIP2;5 (right) at 3 Å steps corresponding to the pore shape in **(B)**. Pore axis (X-Coord): the position along the pore axis is shown as x-coordinate in Å. Dia (Ang): pore diameter value in Å.

Tertiary protein structure predicted for TIPs showed six conserved transmembrane domains. However, the three dimensional geometry of the pore structure obtained with PoreWalker software (Pellegrini-Calace et al., 2009) showed substantial variation in pore size and constrictions in the pore. The tertiary structure and pore morphology for GmTIP1;5 and GmTIP2;5 are shown in **Figure 3**.

**FIGURE 4:**
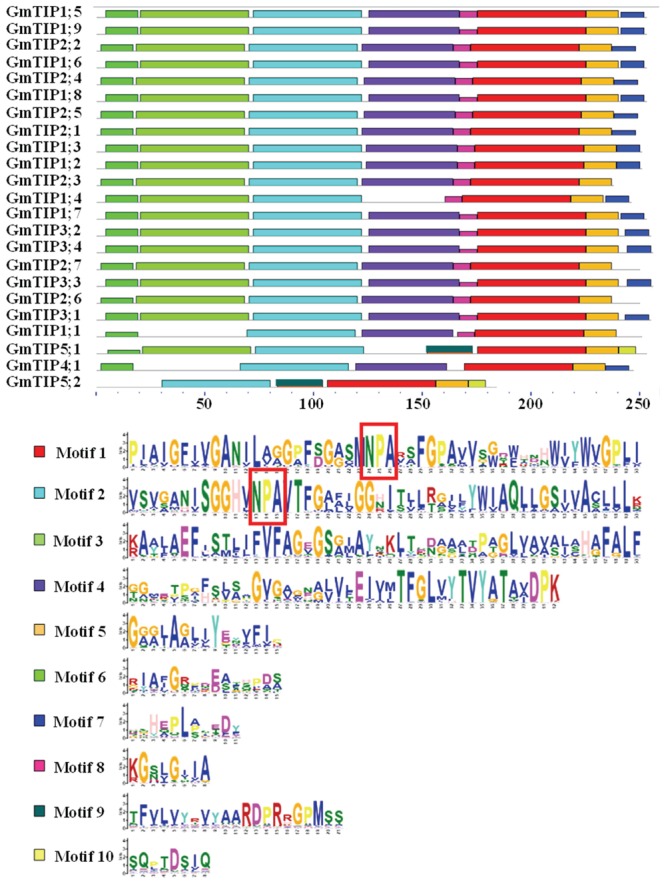
**Identification and distribution of conserved motifs in soybean TIP protein sequences.** Distribution of conserved motifs in soybean TIP members. All motifs were identified by Multiple EM for Motif Elicitation (MEME) using the complete amino acid sequences of GmTIP proteins. Different motifs are indicated by different colored boxes numbered 1–10. The annotation of each motif is listed at the bottom. Motif 1 and motif 2 contain the NPA domain.

The characterization of these aquaporins as TIP genes was based on predicted amino acid similarities with known TIPs from other plant species, with location in the tonoplast of the corresponding proteins needing experimental verification. However, the predicted localization of members of the GmTIP subfamily was very diverse, including cytosol, plasma membrane, endoplasmic reticulum, vacuole, mitochondria, and chloroplast (Zhang et al., 2013). We further searched for conserved motifs in GmTIP proteins with the MEME program to gain additional insights into their diversity. As shown in **Figure 4**, 10 conserved motifs designated as motif 1 to motif 10 were found. All GmTIPs possess motifs 1, 2, and 5. Most of the GmTIPs contain motifs 1 to 8, whereas motif 9 and motif 10 were exclusively present in the GmTIP5 subgroup. Motif 3 was not present in GmTIP1;1, GmTIP4;1, and GmTIP5;2. Motif 4 was not present in GmTIP1;4, GmTIP5;1, and GmTIP5;2.

### Differential Expression of Soybean TIPs in Soybean Tissues and Under Abiotic Stress Conditions

**FIGURE 5:**
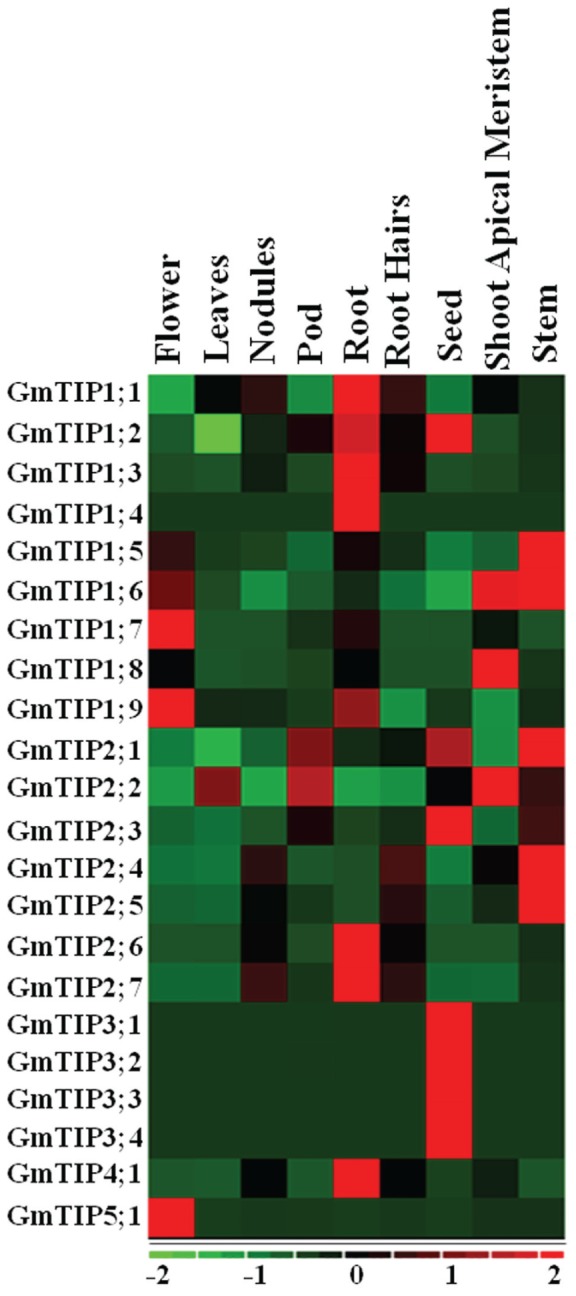
**Heatmap of expression profiles of the soybean GmTIP gene family in nine tissues.** Relative tissue expression levels of GmTIPs based on RNA-seq data were used to construct the expression patterns of soybean genes. The expression data (Reads Per Kilobase Million) values were median-centered and normalized for each gene in different tissue before transforming to color scale. The color bar at the bottom shows the range of expression values from highest expression level (red) to lowest expression level (green), 0 is the median expression level (Black).

Very diverse expression patterns for GmTIPs were observed in the transcriptome data representing nine different tissues, namely flower, leaf, nodule, pod, root, root hair, seed, shoot apical meristem, and stem tissue (Libault et al., 2010). Most of the GmTIPs showed tissue specific expression (**Figure 5**). For example, *GmTIP1;1, 1;2, 1;3, 1;4, 2;6, 2;7*, and *4;1* were highly expressed in root, but have relatively lower expression levels in other tissues. Similarly, *GmTIP1;5, 1;6, 2;4*, and *2;5* were highly expressed in stem, but have a relatively lower expression level in other tissues. No gene showed higher expression levels in leaves and root hairs, while most phylogenetically paired genes showed similar expression patterns. The GmTIP3 subgroup was only expressed in seed tissue, although this pattern broadened under stress conditions (see below). Interestingly, two phylogenetic gene pairs displayed different expression patterns (*GmTIP2;1/GmTIP2;2, GmTIP1;8/GmTIP1;9*), indicating neofunctionalization.

**FIGURE 6:**
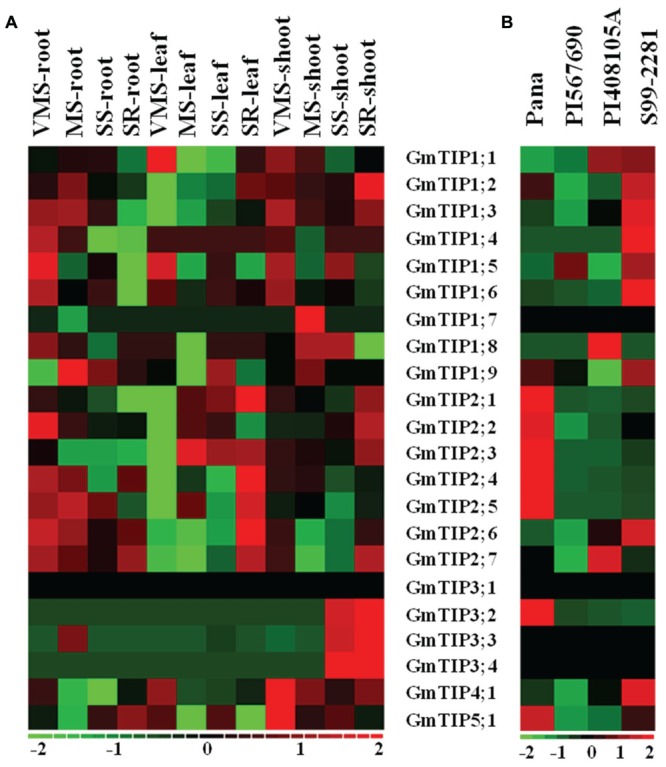
**Expression profiles of soybean TIP genes under different abiotic stress conditions or in different germplasms. (A)** Heatmap representation of expression patterns of soybean TIP genes across the root, leaf, and shoot tissue in Williams 82 under varying water-deficit stress conditions (VMS: very mild stress; MS: mild stress; SS: severe stress; SR: water recovery after severe stress). **(B)** Expression profiles of the soybean TIP genes in leaves of Pana (fast wilting, drought vs. control), PI 567690 (slow wilting, drought vs. control), PI 408105A (flooding tolerant, flooding vs. control), S99-2281(flooding sensitive, flooding vs. control). The expression data values were median-centered and normalized for each gene before transforming to the color scale (log2-transformed ratios). The color bar at the bottom shows the range of expression values from increased expression (red) to decreased expression (green), 0 means no gene expression pattern changed (Black).

The tissue specific expression pattern of GmTIPs was investigated in the Williams 82 genotype under varying water-deficit conditions (**Figure 6A**). *GmTIP3;2, 3;3*, and *3;4* were up-regulated in shoots under serious drought condition, even after water-recovery, and down-regulated in all other tissues and conditions. Interestingly, the whole GmTIP2 subfamily was down-regulated under very mild drought stress in leaf tissue and up-regulated after severe drought stress and water-recovery, except one (*GmTIP2;2*). These results indicated that a gene subfamily may share similar regulatory elements, and also have similar physiological functions. Most of the root-specific expressed genes were induced under varying water-deficit conditions.

To further investigate the expression profiles of TIP genes under drought and flooding conditions, the expression patterns of TIP gene families were extracted from two RNA-seq datasets (Prince et al., 2015 and unpublished data) (**Figure 6B**). PI 567690 is a slow wilting soybean line, and Pana is a fast wilting line under drought conditions (Pathan et al., 2014; Mutava et al., 2015). *GmTIP2;1, 2;2, 2;3, 2;4, 2;5, 3;2*, and *5;1* mRNA levels were induced in leaves of Pana, but decreased in leaves of PI 567690 after drought stress. PI 408105A is a flooding tolerant line, and S99-2281 is a flooding sensitive line (Mutava et al., 2015). *GmTIP1;2, 1;3, 1;4, 1;5, 1;6, 1;9, 2;6*, and *4;1* mRNA levels were induced in leaves of S99-2281, but decreased or were not induced in leaves of PI 408105A after flooding stress. Conversely, *GmTIP1;8* and *2;7* were induced in PI 408105A and decreased in S99-2281. These results indicated that GmTIP genes could play a role under drought or flooding stress.

The expression pattern of the newly identified *GmTIP5;2* could not be determined because all reads from the above RNA-seq datasets were aligned to the previous version of the *G. max* reference genome, Gmax1.1, and Phytozome v9.0. *GmTIP3;1* was not induced or decreased in any tissues (Williams 82) or PI lines under abiotic stress conditions (**Figures 6A,B**). Also *GmTIP3;1*, *GmTIP3;4*, *GmTIP3;3*, and *GmTIP1;7*, did not show any response to abiotic stress in the PI or cultivar lines (**Figure 6B**).

### Analysis of SNP Variation of the GmTIP Gene Family in 106 Soybean Lines

Single-nucleotide polymorphisms located in the coding regions of GmTIPs were identified to investigate the genetic variation within this gene family in diverse soybean lines (Supplementary Table S4). A total of 81 SNPs were observed in the 23 GmTIP genes. These SNPs exhibited an uneven distribution: the GmTIP5 subfamily contains the highest number of SNPs compared to other subfamilies, whereas no SNPs were found in GmTIP2;1 and GmTIP2;3. Out of 81 SNPs, one SNP located in GmTIP5;1 generates a premature stop codon. Thirty-six SNPs were non-synonymous and were distributed in the coding regions of 14 GmTIPs. Some deletions were found in non-synonymous SNP locations, which cause protein-coding changes. Not a single non-synonymous SNP was identified within the remaining seven genes (*GmTIP1;2*, *GmTIP1;5, GmTIP1;9, GmTIP2;4, GmTIP2;5, GmTIP2;6*, and *GmTIP4;1*). SNPs unique to *G. soja* lines (PI# highlighted red) are summarized in Supplementary Table S4. Only 18 unique SNPs were identified in seven *G. soja* lines, and eight out of these 18 SNPs were non-synonymous. The pattern of SNP distributions correlates well with the phylogenetic distribution of TIPs (**Figure 1**; Supplementary Table S2). This sequence information enabled us to identify novel GmTIP alleles from soybean lines that will serve as valuable breeding resources.

**FIGURE 7:**
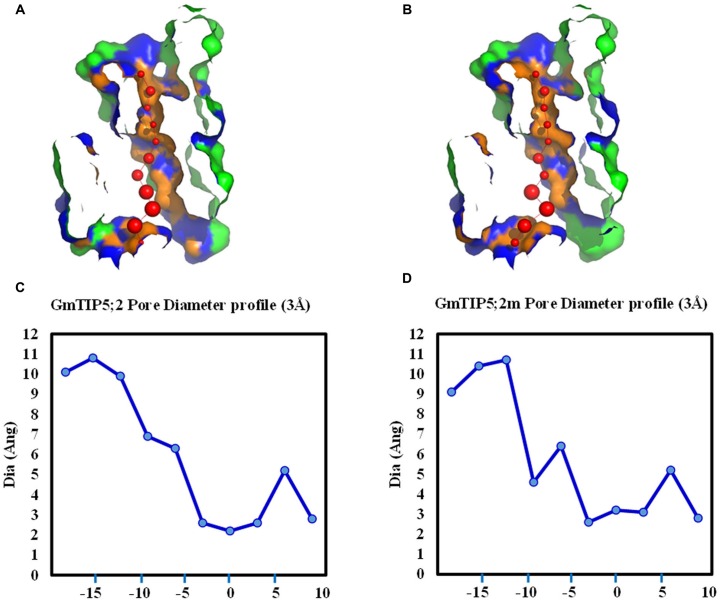
**Variation in pore morphology of GmTIP5;2 and GmTIP5;2m.** GmTIP5;2m contains two amino acid changed (148: A<>G; 159: T<>R) due to two SNP (Gm12_895269 and Gm_895302). **(A)** and **(B)** Cross sections of the proteins showing pore of GmTIP5;2 **(A)** and GmTIP5;2m **(B)**. **(C)** and **(D)** Pore diameter profile of GmTIP5;2 **(C)** and GmTIP5;2m **(D)** at 3 Å steps. Pore axis (X-Coord): the position along the pore axis is shown as x-coordinate in Å. Dia (Ang): pore diameter value in Å.

Genetic variations were further investigated in four soybean slow wilting lines (PI 471938, PI 416937, PI 567690, and PI 567731) to establish associations between SNPs and the slow wilting trait. However, no unique SNPs were identified within coding sequences in these four slow wilting lines as compared with other non-slow wilting lines. The effects of non-synonymous SNP on the pore shape were further analyzed in GmTIP5;1 and GmTIP5;2, which have more non-synonymous SNPs in the protein coding region than other GmTIPs. Interestingly, we find that two non-synonymous SNPs (Gm09_41743311 and Gm09_41743354) in GmTIP5;1 do not influence the channel 3D shape. However, three non-synonymous SNPs (Gm12_895269, Gm12_895302, and Gm12_895476) in GmTIP5;2 have an effect on the pore shape. Different pore diameter profiles were obtained between Williams 82 genotype and some of the other sequenced genotypes (**Figure 7**). For example, the structure of GmTIP5;2 in PI 567690 was different than that in Williams 82. These results indicate that the slow wilting trait in these lines may be governed by different molecular mechanisms, such as GmTIP expression level differences or is due to their different 3D structures.

### Cloning and Functional Characterization of *GmTIP1;5* and *GmTIP2;5* in *Xenopus* Oocytes

**FIGURE 8:**
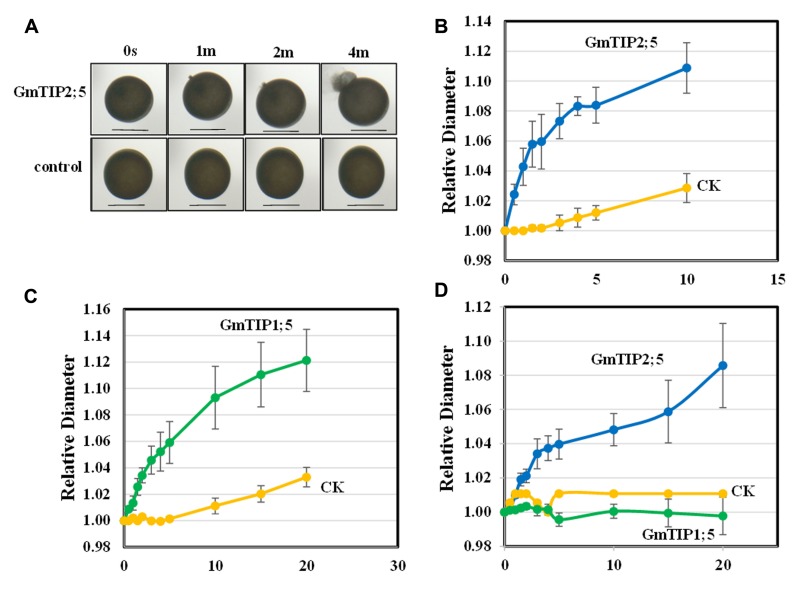
**Oocyte Swelling Assays with GmTIP1;5 and GmTIP2;5.** The relative diameter of GmTIP-expressing oocytes following exposure to hypoosmotic or boron-containing isosmotic solutions was measured to characterize the osmotic permeability of GmTIP1;5 and GmTIP2;5-containing membranes. **(A)** Water transport assay with *Xenopus leaevis* oocytes in hypoosmotic solution showed that GmTIP2;5-mediated water uptake leads to rapid swelling and bursting (top) compared to the control (bottom). Scale bars = 1 mm. **(B)** Rate of oocyte swelling in hypoosmotic solution in mock-injected controls (yellow line) vs. GmTIP2;5-expressing oocytes (blue line). Oocytes were observed to burst after 10 min. **(C)** Rate of oocyte swelling in hypoosmotic solution in mock injected controls (yellow line) vs. GmTIP1;5-expressing oocytes (green line). Results are reported as means ± SEM (*N* = 5 oocytes). **(D)** Rate of oocyte swelling in isosmotic boric acid solution in mock injected controls (yellow line) vs. GmTIP2;5-expressing oocytes (blue line) and GmTIP1;5 (green line). The volume of each oocyte was measured for 20 min or until it burst.

No non-synonymous SNP was found in *GmTIP1;5* and *GmTIP2;5* in 106 soybean germplasm; however, they were shown to be differentially expressed in response to drought (*GmTIP1;5* and *GmTIP2;5*) and flooding (*GmTIP1;5*) in different varieties of soybeans with contrasting phenotypes and were mainly expressed in stem where they could be highly important in water transport during abiotic stress. Further in-depth analysis of *GmTIP1;5* and *GmTIP2;5* using *X. laevis* oocytes was performed to establish a link between differential expression patterns and trait development. GmTIP2;5 and GmTIP1;5-expressing oocytes showed a rapid rate of swelling for the first 5 min after being subjected to a hypoosmotic solution (**Figures 8A–C**). After 5 min, the rate of swelling in *GmTIP2;5* expressing oocytes declined as they began to burst. In contrast, *GmTIP1;5* expressing oocytes continued to increase in diameter at a rate greater than the controls for 20 min. GmTIP2;5-expressing oocytes swelled more rapidly than GmTIP1;5-expressing oocytes during the first 5 min. Mock-injected oocytes displayed a slower rate of swelling, probably due to diffusion of water across the plasma membrane. It was concluded from these results that GmTIP2;5 and GmTIP1;5 facilitate the transport of water and are aquaporins.

Some aquaporins transport boron and other solutes in addition to water (Durbak et al., 2014). To test if these GmTIPs can transport boron, we applied an isoosmotic solution containing 200 mM boric acid in place of 96 mM NaCl. The GmTIP2;5-expressing oocytes showed significantly increased swelling with boric acid compared to the control (**Figure 8D**). In contrast, control and GmTIP1;5-expressing oocytes did not swell. These results indicated that GmTIP2;5 can transport water as well as boric acid.

## Discussion

### GmTIPs Play Important Roles in Abiotic Stress

Here, 23 full-length aquaporin-coding sequences belonging to the TIP subfamily were identified in the soybean genome. The number of TIPs in the soybean genome is much higher than those identified in species like *Arabidopsis*, maize, rice, and sorghum (Regon et al., 2014). Several studies have shown differential expression of plant aquaporins in response to environmental stresses in several different species (Alexandersson et al., 2005; Zhu et al., 2005; Ligaba et al., 2011; Cohen et al., 2013; Li et al., 2014). Therefore, it is important to investigate the interaction between the expression of GmTIPs and abiotic stress. This study explored the expression patterns of GmTIP genes in soybean PI 567690 and Pana lines. The soybean genotype PI 567690 exhibits significantly lower wilting and less yield loss under drought condition than the elite cultivar Pana (Pathan et al., 2014). The difference in gene expression between PI 567690 and Pana had been investigated by RNA-seq (Prince et al., 2015), and our preliminary data indicated that the PI 567690 genotype has a limited transpiration response when exposed to a high vapor pressure deficit (unpublished data). In the present analysis, we found that the expression level of seven TIP genes was reduced in slow-wilting soybean lines but increased in the fast-wilting soybean lines after drought stress (**Figure 6B**). At the same time, the expression level of eight different TIP genes was down-regulated or unchanged in the waterlogging tolerant varieties, but upregulated in the waterlogging susceptible varieties. Only one gene was found to be up-regulated in slow-wilting lines under drought conditions, and only three genes were found to be up-regulated in the water-logging tolerant lines. These results are consistent with the hypothesis that due to lessened water transport in soybean leaves, the genotype PI 567690 exhibited more resistance to drought stress. We therefore conclude that GmTIPs may play an important role in soybean both in drought and flooding tolerance. However, the gene regulation patterns in root tissue of water-logging resistant lines should be further explored.

The expression patterns of GmTIPs under a given condition varied among different tissues and was complex (**Figures 6A,B**). *GmTIP1;5* and *GmTIP2;5* showed a higher expression in stem, but lower expression in other tissues. Interestingly, *GmTIP2;5* transcript was up-regulated under varying water-deficit stress in root, but down-regulated after water-recovery. This is the TIP that showed a very rapid water uptake in *Xenopus* oocytes. *GmTIP1;5*, which showed a slower, but steady uptake in *Xenopus* oocytes, was mainly induced under the VMS condition in root, leaf and shoot, not in other water-deficit conditions. Furthermore, *GmTIP1;5* is the only one that showed upregulation in slow wilting lines under drought condition among all *TIP* genes. In addition, only one synonymous SNP was located in the coding sequences of *GmTIP1;5* and two synonymous SNPs were located in the coding sequence of *GmTIP2;5*. Therefore, observed variations in gene expression seem to have prominent roles in functional variation. Further investigation of the water transport function of these two genes in soybean would help to establish a link between differential expression patterns and trait development.

To gain further insight into the possible physiological functions of the members of this large family and aid the development of genetic engineering in soybean, the *TIP* that directs tissue-specific expression pattern and functions in water transport would be highly desirable. Overall, a more comprehensive mechanism will emerge as multiple aquaporin transport functions and integration of the different stress signals are determined at the whole plant level. Further characterization of the GmTIP genes involved in abiotic stress resistance genotypes may aid in developing tolerant germplasm and cultivars.

### Water Permeability of GmTIPs

All *G. max* TIP aquaporins showed the canonical double NPA motif and a group ar/R SF that are conserved across different species. These results indicate that GmTIPs may play similar roles in regulating water absorption. As such, it is important to validate representative candidate genes for water transport by using other methods such as heterologous expression in *Xenopus* oocytes. Basic GmTIP gene family information and phylogenetic sequence analysis in the present study provide a list of candidate genes that may play important roles in soybean drought and flooding tolerance. The function of two of these GmTIP genes has been demonstrated to be involved in water transport. However, AQPs are not only water transporters but also solute transporters (Maurel et al., 2008). Most AQPs can transport glycerol, urea, boric acid or arsenic as we showed with boron and GmTIP2;5. Yet even though a protein has been predicted to be an aquaporin, it may not transport water. Thus GmTIP candidate genes must be functionally characterized.

Aquaporins can be further functionally characterized with transport inhibitors. One such inhibitor, AgNO_3_ or silver sulfadiazine, has been reported to inhibit aquaporin water transport by binding to cysteine or histidine residues, resulting in blockage of the pore (Daniels et al., 1996; Ishibashi et al., 1997; Niemietz and Tyerman, 2002). Interestingly, the transpiration rate of the slow-wilting cultivar PI416937 is insensitive to silver treatment, and only a small change in AQP abundance following silver treatment was found in this line as compared to sensitive genotypes (Sadok and Sinclair, 2010b). All these results suggest that low AQP abundance may underlie the low leaf hydraulic conductivity of PI 416937 and its limited transpiration rates under high vapor pressure deficit (Sadok and Sinclair, 2010a, 2012; Devi et al., 2015, 2016). The effects of silver ions on water transport in *Xenopus* oocytes expressing GmTIP1;5, GmTIP2;5, or other AQPs could help us to identify key candidate genes linked with the soybean slow-wilting trait. For example, we hypothesize that *GmTIP5;1* would be a good target gene for engineering reduced gene expression for improved water stress tolerance, since under severe water-deficit conditions *GmTIP5;1* was induced in shoot and leaf tissues but showed decreased expression in the slow wilting line and flooding tolerant lines under stress conditions.

### Utilization of Natural Variation of GmTIPs

Analysis of whole genome resequencing data provides an immense opportunity to mine natural variants in diverse germplasm (Patil et al., 2016; Valliyodan et al., 2016). The soybean germplasm, both wild and cultivated species, provides a wide range of abiotic stress tolerance. This study initiated characterization of GmTIPs by summarizing and analyzing all available SNP information, including whether SNPs lead to synonymous or non-synonymous substitutions, in 106 soybean lines (Valliyodan et al., 2016). These 106 soybean lines represent diverse lines (wild types, landraces and elite lines), including four slow wilting soybean lines (PI 416937, PI 471938, PI 567690, and PI 567731). Aside from the expression level, genetic variation (natural or induced) also may impact the functionality of aquaporin genes and cause a variation in phenotypes. To our knowledge, this is the first report of natural variation in soybean for TIP genes. With this information, the correlation between the SNP haplotype and the phenotype (slow wilting score) for these 106 lines can be calculated to further characterize the association between the GmTIP genetic background and the slow-wilting trait. Furthermore, the association analysis can be extended to the whole MIP gene family and the gene promoter region to better understand the mechanism of slow wilting at the genomic level.

It has been reported that plant plasma membrane aquaporins are deactivated by dephosphorylation under conditions of drought stress, or by protonation of a conserved histidine residue following a drop in cytoplasmic pH due to anoxia during flooding (Tournaire-Roux et al., 2003; Törnroth-Horsefield et al., 2006). Analyzing the detailed effects of all non-synonymous SNPs on the existing crystal structure of plant AQPs (for examples: AtTIP2;1, SoPIP2;1) (Törnroth-Horsefield et al., 2006) in some interesting soybean lines may enable us to better understand the structure changes and mechanisms of water transport regulation.

## Conclusion

In this study, the soybean TIP gene family was identified based on the new soybean genome annotation. Twenty-three TIP members were assigned to five subfamilies based on sequence similarity and phylogenetic relationship. The modulation of expression profiles of these 23 GmTIP genes was examined with deep transcriptome sequencing under drought or flooding conditions and during plant development. The potential roles of these genes in transport of substrates were also discussed. The identified natural variations in this gene family from 106 soybean germplasms will benefit future gene functional analysis and utilization. We also demonstrated that GmTIP1;5 and GmTIP2;5 can function as water transporters in oocytes. All results presented here represent an important resource for designing experiments for functional validation of candidate genes in plant development and abiotic stress responses, and for developing soybean germplasm with improved abiotic stress tolerance.

## Author Contributions

LS and RD designed the experiments, analyzed data, and prepared the manuscript. NN, SMP, LS and WG worked on the oocytes experiments. GP, RD, and BV analyzed the natural variation SNP data. RD and LS worked on defining pore-lining residues and solute prediction. SJP and RM were involved in the gene expression pattern analysis. HN conceived and supervised the project. All authors have read, revised, and approved the manuscript.

## Conflict of Interest Statement

The authors declare that the research was conducted in the absence of any commercial or financial relationships that could be construed as a potential conflict of interest.

## Footnotes

1 https://phytozome.jgi.doe.gov

2 http://gsds.cbi.pku.edu.cn/

3 http://topcons.cbr.su.se/

4 http://swissmodel.expasy.org/interactive

5 http://www.ebi.ac.uk/thornton-srv/software/PoreWalker/

6 http://chibba.agtec.uga.edu/

7 http://snpeff.sourceforge.net
