# Supplementary material for: Soybean TIP Gene Family Analysis and Characterization of GmTIP1;5 and GmTIP2;5 Water Transport Activity
Source: Front Plant Sci. 2016 Oct 21;7:1564. doi: 10.3389/fpls.2016.01564 (PMC5073556; doi:10.3389/fpls.2016.01564)
Supplement: Supplementary file 3 [file Table_3.DOCX]

**Table S3** Details of NPA domains Ar/R selectivity filter**,** Froger’s residues and the spacing between NPA domains present in soybean tonoplast intrinsic proteins

| **Sr. No** | **Gene_Id** | **Manually assigned features based on protein sequence alignment** | | | | | | | | | | | |
| --- | --- | --- | --- | --- | --- | --- | --- | --- | --- | --- | --- | --- | --- |
|  |  | **NPA (LB)** | **NPA (LE)** |  | **Ar/R filters** | | | | **Froger’s residues** | | | | |
|  |  |  |  | **Spacing between NPA domains** | **H2** | **H5** | **LE1** | **LE2** | **P1** | **P2** | **P3** | **P4** | **P5** |
| 1 | GmTIP1;1 | NPA | NPA | 113 | H | V | A | A | T | S | A | Y | W |
| 2 | GmTIP1;2 | NPA | NPA | 110 | H | I | A | V | T | T | A | Y | W |
| 3 | GmTIP1;3 | NPA | NPA | 110 | H | I | A | V | T | T | A | Y | W |
| 4 | GmTIP1;4 | NPA | NPA | 104 | H | I | A | V | T | S | A | Y | W |
| 5 | GmTIP1;5 | NPA | NPA | 111 | H | I | A | V | T | S | A | Y | W |
| 6 | GmTIP1;6 | NPA | NPA | 111 | H | I | A | V | T | S | A | Y | W |
| 7 | GmTIP1;7 | NPA | NPA | 111 | H | I | A | V | T | C | A | Y | W |
| 8 | GmTIP1;8 | NPA | NPA | 111 | H | I | A | V | T | S | A | Y | W |
| 9 | GmTIP1;9 | NPA | NPA | 111 | H | I | A | V | T | S | A | Y | W |
| 10 | GmTIP2;1 | NPA | NPA | 110 | H | I | G | R | T | S | A | Y | W |
| 11 | GmTIP2;2 | NPA | NPA | 110 | H | I | G | R | T | S | A | Y | W |
| 12 | GmTIP2;3 | NPA | NPA | 110 | H | I | G | R | T | S | A | Y | W |
| 13 | GmTIP2;4 | NPA | NPA | 111 | H | I | G | R | T | S | A | Y | W |
| 14 | GmTIP2;5 | NPA | NPA | 111 | H | I | G | R | T | S | A | Y | W |
| 15 | GmTIP2;6 | NPA | NPA | 110 | H | I | G | R | T | S | A | Y | W |
| 16 | GmTIP2;7 | NPA | NPA | 110 | H | I | G | R | T | S | A | Y | W |
| 17 | GmTIP3;1 | NPA | NPA | 111 | H | I | A | R | T | A | A | F | W |
| 18 | GmTIP3;2 | NPA | NPA | 111 | H | I | A | R | T | A | A | F | W |
| 19 | GmTIP3;3 | NPA | NPA | 111 | H | I | A | L | T | A | S | F | W |
| 20 | GmTIP3;4 | NPA | NPA | 111 | H | I | A | L | T | A | S | F | W |
| 21 | GmTIP4;1 | NPA | NPA | 111 | H | I | A | R | S | S | A | Y | W |
| 22 | GmTIP5;1 | NPA | NPA | 110 | S | V | G | C | V | A | A | Y | W |
| 23 | GmTIP5;2 | NPA | NPA | 110 | S | V | G | C | L | A | A | Y | W |
